# Neurodevelopmental outcome in infants with neonatal encephalopathy receiving hydrocortisone during therapeutic hypothermia: follow-up of the extended-CORTISoL trial

**DOI:** 10.1038/s41372-025-02428-5

**Published:** 2025-09-22

**Authors:** Kata Kovacs, Eniko Szakmar, Marianna Dobi, Zsuzsanna Varga, Unoke Meder, Attila J. Szabo, Patrick J. McNamara, Miklos Szabo, Agnes Jermendy

**Affiliations:** 1 Department of Neonatology, Pediatric Center, Semmelweis University, Budapest, Hungary; 2 Departments of Pediatrics and Internal Medicine, University of Iowa, Iowa city, IA USA

**Keywords:** Outcomes research, Risk factors

## Abstract

**Objective:**

To investigate neurodevelopmental outcome after hydrocortisone (HC) supplementation in infants with neonatal encephalopathy (NE) and hypotension.

**Study design:**

Fifty-five infants with volume-resistant hypotension during hypothermia were enrolled between 2016 and 2020. Eligible patients were randomly assigned to receive 0.5 mg/kg HC every 6 h or placebo along with standard dopamine treatment. Composite adverse outcome was defined as death or neurodevelopmental impairment, ascertained using the Bayley-II test.

**Result:**

At median 20 months of age, death, or severe neurodevelopmental impairment occurred in 40% in the HC group, compared to 18% in the placebo group (*p* = 0.13). Multiple logistic regression analysis showed that for every 1 mg/kg increase in cumulative HC dose, the odds of adverse cognitive outcome increased by 16% (95% CI 1.01–1.37; *p* = 0.04).

**Conclusion:**

The composite outcomes were similar in the HC-treated and placebo groups, however cumulative HC dose was associated with adverse cognitive outcome in infants with NE and hypotension.

**Clinical trial registration:**

Hydrocortisone treatment in systemic low blood pressure during hypothermia in asphyxiated newborns (CORTISoL), ClinicalTrials.gov ID: NCT02700828 https://clinicaltrials.gov/ct2/show/NCT02700828, date of registration: 2016-02-24.

## Introduction

Hydrocortisone (HC) has been used as a rescue therapy in neonates with vasopressor-resistant hypotension, particularly when an underlying adrenal dysfunction is considered [1–3]. Previously, we have demonstrated that supplemental HC for suspected relative adrenal insufficiency is effective in raising blood pressure in infants with neonatal encephalopathy (NE) in a randomized, controlled trial (CORTISoL study; HC treatment in systemic low blood pressure during hypothermia in asphyxiated newborns) [4]. Despite its efficiency, steroid supplementation should be considered carefully in neonatal populations due to its potential negative effects on neurodevelopment [5]. Published data on the relationship between long-term outcomes and HC treatment are conflicting, and it remains unknown whether HC independently affects neurodevelopmental impairment. Some studies on preterm infants have reported a lower or similar rate of adverse neurodevelopmental outcome after HC therapy [6–9], while others showed a higher rate [10, 11], compared to infants who did not receive HC. It is uncertain whether this is due to treatment effects or the severity of the underlying illness. Finally, most neurodevelopmental observations relate to HC use in preterm infants; therefore, the impact of HC therapy on neurodevelopmental outcomes in infants with NE remains a knowledge gap.

In this study, we report the neurodevelopmental outcomes of an extended cohort of patients from the randomized controlled CORTISoL study at two years of age. We hypothesized that neurodevelopmental outcome is not impacted by early, HC supplementation.

## Methods

### Patient population

This secondary analysis was performed using data collected from an extended cohort of the CORTISoL study, which was a single-center, double-blinded, randomized, placebo-controlled, pragmatic trial of HC supplementation used for systemic hypotension during hypothermia treatment in infants with NE. The study was conducted at the NICU of the Pediatric Center, Semmelweis University. Patients were enrolled between February 2016 and February 2020. Ethical permission was obtained from the Scientific and Medical Research Council Ethics Committee of Hungary (5705-1/2016/EKU). The study was registered with ClinicalTrials.gov under the NCT02700828 identification number.

Inclusion criteria were as follows (i) ≥36 weeks of gestational age; (ii) whole-body hypothermia treatment (as described by Azzopardi et al. [12]); and (iii) presence of systemic hypotension (defined as a mean arterial blood pressure < gestational age in weeks) during hypothermia treatment [13]. Infants were excluded if they had received (i) vasopressor-inotrope therapy before randomization, (ii) presented with congenital abnormalities or genetic disorders, or (iii) their hematocrit level was <35%. Signed informed parental consent was obtained in all cases.

In brief, patients were randomly assigned to receive 0.5 mg/kg bolus HC (Solu-Cortef, HC-sodium-succinate, Pfizer Kft., Budapest, Hungary) every 6 h (HC group) or the same amount of placebo (isotonic saline). Simultaneously, irrespective of group allocation, 6 µg/kg/min intravenous dopamine infusion was initiated at the time of hypotension in all patients to ensure timely management of low blood pressure. Other details of the randomization procedure, drug preparation, management of systemic hypotension and data collection are described in the original article, published in 2019 [4]. As part of the routine NE management at our unit, infants had a brain MRI examination as soon as they reached clinical stability. Further details of clinical and cardiovascular care and neuromonitoring are reported in the Online Supplementary Material.

### Neurodevelopmental follow-up

The secondary endpoint of the original CORTISoL study was death or adverse neurodevelopmental outcome at two years of age using the Bayley Scales of Infant Development, Second Edition (Bayley II). Adverse neurodevelopmental outcome was defined as a score of <70 on either the Mental Development Index (MDI) or the Psychomotor Development Index (PDI). Outcome was assessed by a trained clinical psychologist (ZsV), blinded to the randomization and clinical care. For infants who could not be tested properly with Bayley II due to cerebral paresis or lack of cooperation, motor and cognitive categories were assigned, with scores at the lower boundary of the respective category (85 [−1SD]—mild delay, 70 [−2SD]—moderate delay, and 50 [−3SD]—severe delay, and 45 for those with severe CP). A sample size calculation was performed specifically for neurodevelopmental outcomes in the CORTISoL study. Using a non-inferiority study design, sample size calculation suggested that a total of 56 patients—28 in each treatment arm—was needed to show that patients receiving HC had similar mental or motor developmental scores compared to patients receiving placebo, with a power of 80%, and using a non-inferiority margin of 14%. Sample size calculation was based on our previous observational data and population statistics of Bayley II tests [14, 15].

At the time of the neurodevelopmental follow-up examination, parents completed a national socioeconomic status questionnaire that has been used previously in the Hungarian population of infants with NE [16]. The parents’ educational attainment was divided into three categories: (1) parents with elementary or technical school education; (2) at least one of the parents had a high-school diploma; (3) at least one of the parents had a university degree. Literacy environment was assessed by the number of books, specifically, >150 or <150 books in the household.

### Statistical analysis

Descriptive statistics were expressed as median and interquartile range, or number and percentage. Non-parametrical statistical tests (Chi-square test, Bernard exact test, Mann–Whitney U test) were used as appropriate to compare clinical characteristics and outcomes between treatment groups. Patient outcomes were analyzed according to intention-to-treat, comparing death or adverse neurodevelopmental outcome rates in the two study groups (binary outcome). Continuous Bayley scores are also reported in the tables. Multiple regression modeling was performed to predict adverse outcomes. Receiver Operator Characteristic (ROC) analysis was performed to determine the cut-off value of cumulative HC dose (mg/kg), which may have an influence on any adverse outcome.

Statistical analysis was performed using R Statistical Software 4.1.3 (R Foundation for Statistical Computing, Vienna, Austria) and IBM SPSS Statistics 28.0.1.0 (IBM Corp, Armonk, New York). The level of significance was set at 0.05.

## Results

During the 4-year study period, 225 infants with NE were treated with therapeutic hypothermia at the level IV NICU of the Pediatric Center, Semmelweis University, of whom 57 were enrolled in the current study (Supplemental Fig. 1). According to the randomization, 26 infants were assigned to the HC group and 31 infants to the placebo group. Two patients were excluded from the analysis after randomization due to later identification of confounding diagnoses made.

Baseline clinical characteristics of the study population were comparable between the HC and placebo groups (Table 1). The initial random serum cortisol levels were low in both groups, of which 76% of the samples were below 15 µg/dL, the threshold of relative adrenal insufficiency [17, 18] (HC group 77%, placebo group 76%). HC supplementation was started at a median of 11.5 [6.0; 20.5] h of postnatal life, and lasted for a median of 2.9 [1.6; 4.9] days. Cumulative HC dose was median 5.8 [5.3; 6.6] mg/kg in the HC group. Regarding the cardiovascular management, duration of inotrope therapy as well as peak dose of dopamine treatment were lower in the HC group compared to the placebo group (Table 2).

**Table 1:** Clinical characteristics of the study population according to the intention-to-treat analysis.

Parameters	HC group (*n* = 26)	Placebo group (*n* = 29)	*p* value
Gestational age (weeks)	39 [38; 40]	39 [38; 40]	0.98
Birth weight (g)	3505 [2775; 3660]	3300 [3000; 3750]	0.83
Gender (female/male), *n* (%)	7 (27)/19 (73)	15 (52)/14 (48)	0.10
Mode of delivery (vaginal/caesarian section), *n* (%)	16 (62)/10 (38)	15 (52)/14 (48)	0.59
Maternal age (years)	30 [29; 33]	30 [26; 37]	0.56
Apgar scores			
1 min	3 [1; 5]	3 [2; 5]	0.85
5 min	5 [4; 6]	6 [4; 7]	0.88
10 min	6 [5; 7]	7 [5; 8]	0.27
First blood gas analysis			
pH	7.0 [6.9; 7.1]	7.0 [6.8; 7.1]	0.84
pCO_2_ (mmHg)	70 [52; 79]	60 [45; 75]	0.12
Base deficit (mmol/L)	−16.0 [−21.1; −13.0]	−16.0 [−21.5; −12.0]	0.98
HCO_3_^−^ (mmol/L)	15.1 [11.8; 16.1]	10.8 [9.2; 17.3]	0.09
Serum lactate (mmol/L)	15.0 [12.8; 17.0]	14.7 [11.6; 18.2]	0.81
Glucose (mmol/L)	5.5 [3.6; 7.5]	5.4 [4.1; 7.9]	0.76
Age at the beginning of hypothermia treatment (h)	2.6 [1.7; 3.5]	2.6 [1.7; 3.9]	0.74
Age at randomization (h)	11.5 [6.0; 20.5]	14.5 [7.9; 21.1]	0.50
Baseline serum cortisol concentration immediately before randomization (μg/dL)	3.3 [2.5; 14.5]	3.1 [2.3; 15.0]	0.95
Unilateral adrenal bleeding, *n* (%)^a^	1 (4)	2 (7)	1.00
SNAP-II score before randomization	29 [20; 42]	36 [20; 43]	0.45
NE severity, *n* (%)^b^			
mild	7 (27)	13 (45)	0.37
mild-moderate	6 (23)	6 (21)	
moderate	7 (27)	5 (17)	
moderate-severe	4 (15)	1 (3)	
severe	2 (8)	4 (14)	
aEEG score within the first 6 h^c^	2.75 [1.75; 4.0]	2.0 [1.0; 3.0]	0.17

Data shown as median and interquartile ranges. See text for details.

*aEEG* amplitude integrated electroencephalogram, *HC* hydrocortisone, *NE* neonatal encephalopathy, *SNAP-II* score for neonatal acute physiology, second edit.

^a^*n* = 54 (HC group *n* = 26; Placebo group *n* = 28).

^b^Severity of neonatal encephalopathy was determined after rewarming, based on the overall aEEG background activity, occurrence of clinical and/or electrophysiological seizures, MRI results and clinical symptoms of encephalopathy.

^c^aEEG background activity was scored as follows: continuous normal voltage (score 1), discontinuous normal voltage (score 2), burst suppression (score 3), continuous low voltage (score 4), and flat trace (score 5).

**Table 2:** Cardiovascular management of the study population according to the intention-to-treat analysis.

Parameters	HC group (*n* = 26)	Placebo group (*n* = 29)	*p* value
Cardiovascular management			
Lowest systolic BP (mmHg)	47 [43; 53]	42 [39; 47]	**0.01**
Lowest mean BP (mmHg)	35 [31; 36]	33 [28; 34]	0.055
Lowest diastolic BP (mmHg)	27 [23; 29]	26 [22; 28]	0.43
Heart rate at the time of lowest BP (beats/min)	90 [82; 94]	93 [82; 108]	0.24
Duration of inotrope therapy (h)	59 [21; 88]	91 [68; 111]	**0.003**
Combined inotrope therapy, *n* (%)	1 (4)	7 (24)	0.054
Peak dose of dopamine treatment (µg/kg/min)	6.2 [6.0; 9.9]	12.0 [8.2; 17.6]	**<0.001**
Open-label HC treatment, *n* (%)	4 (15)	12 (41)	**0.042**

Data shown as median and interquartile ranges. See text for details.

Statistically significant P values are set in bold.

*BP* blood pressure, *HC* hydrocortisone.

Brain MRI was done on median postnatal day 5; and a similar pattern of brain injury was described in both groups (Table 3). The overall follow-up rate was 96% (*n* = 53) and patients were examined at a median 20 [19; 22] month of age. Importantly, the rate of composite outcome (death or adverse neurodevelopmental outcome) was similar in the HC and placebo groups: death or severe neurodevelopmental impairment occurred in 10 of 25 infants (40%) in the HC group, as compared with 5 of 28 infants (18%) in the placebo group (*p* = 0.13). In addition, we found no difference in the median MDI and PDI scores between the HC and placebo groups among survivors (Table 3).

**Table 3:** Short- and long-term outcomes of the study population according to the intention-to-treat analysis.

Parameters	HC group (*n* = 26)	Placebo group (*n* = 29)	*p* value
aEEG background activity normalization within 48 h, *n* (%)	18 (69)	20 (77)	0.76
Anticonvulsant therapy, *n* (%)	16 (62)	11 (38)	0.11
Multiorgan failure, *n* (%)	19 (73)	21 (72)	1.00
Anuria/oliguria, *n* (%)	6 (23)	7 (24)	1.00
Antibiotic treatment (day)	2 [1; 4]	2 [1; 6.5]	0.51
Hyperglycemic episodes requiring insulin therapy, *n* (%)	0 (0)	0 (0)	1.00
Gastrointestinal bleeding, perforation, *n* (%)	0 (0)	0 (0)	1.00
Length of hospitalization (day)	11.5 [8.8; 14.3]	9.7 [7.6; 12.3]	0.27
Brain MRI findings, *n* (%)^a^			
Time of MRI scan (day of life)	5.2 [4.5; 6.5]	5.1 [4.4; 6.3]	0.55
Weeke scoring			
Deep grey matter subscore	3 [0; 6]	0 [0; 4]	0.20
White matter/cortex subscore	2 [0; 7]	1 [0; 3]	0.13
Cerebellum subscore	0 [0; 0]	0 [0; 0]	0.87
Intraventricular hemorrhage	6 (23)	3 (12)	0.47
Subdural hemorrhage	11 (42)	8 (31)	0.57
Cerebral sinovenous thrombosis	0 (0)	0 (0)	*–*
Total score	6 [2; 14]	1 [0;5]	0.08
Total score ≥ 1 point	21 (81)	17 (65)	0.35
Bayley II test^b^			
MDI points	89 [71; 99]	85 [75; 94]	0.61
PDI points	86 [46; 100]	85 [70; 102]	0.53
Age at Bayley II test (month)	19.5 [18; 21]	20 [19; 23]	0.19
Socioeconomic background^c^			
Parental educational level			0.40
Elementary or technical school education	2 (9)	6 (24)	
High-school diploma	9 (41)	10 (40)	
University degree	11 (50)	9 (36)	
Number of books <150	12 (55)	16 (64)	0.56
Death, *n* (%)	1 (4)	1 (3)	1.00
Composite adverse outcome, *n* (%)^d^	10 (40)	5 (18)	0.13

Data shown as median and interquartile ranges. See text for details.

Composite adverse outcome was defined as death or Mental Development Index (MDI) and/or the Psychomotor Development Index (PDI) <70 points at the Bayley II test.

*aEEG* amplitude integrated electroencephalogram, *HC* hydrocortisone, *MDI* mental development index, *NE* neonatal encephalopathy, *PDI* psychomotor development index.

^a^*n* = 52 (HC group *n* = 26; Placebo group *n* = 26).

^b^*n* = 51 (HC group *n* = 24; Placebo group *n* = 27).

^c^*n* = 47 (HC group *n* = 22; Placebo group *n* = 25).

^d^*n* = 53 (HC group *n* = 25; Placebo group *n* = 28).

It must be acknowledged that a total of 16 infants received open-label HC therapy due to critical deterioration, irrespective of randomization. Open-label HC was administered more frequently in the placebo group (Table 2). The supplementation was started at a median of 84.6 [23.6; 89.8] h of postnatal life, typically beyond the study period of therapeutic hypothermia, and lasted for a median of 2.3 [1.5; 3.8] days. Cumulative HC dose was median 6.6 [3.0; 9.8] mg/kg. The illness severity score of patients who received open-label HC (Score for Neonatal Acute Physiology, SNAP-II score) was significantly higher compared to those infants who received the blinded, randomized treatment (median 40 [27; 47] vs 27 [20; 40] points, respectively; *p* = 0.03).

As a final step in our analysis, we conducted a detailed assessment of the potential association between cumulative HC dose and neurodevelopmental outcomes within our patient population. We performed multiple logistic regression analyses to ascertain the effect of cumulative HC dose on the likelihood of composite adverse outcome, as well as cognitive and psychomotor outcomes separately, adjusting for clinically relevant confounders (NE severity and parental educational level). Regarding the composite adverse outcome (MDI and/or PDI <70 or death), only NE severity was associated with the long-term outcome (Table 4a). Cumulative HC dose (including open-label HC administration as well) was significantly associated with the long-term cognitive outcome (Table 4b), but not with psychomotor outcome (Table 4c). For every 1 mg/kg increase in cumulative HC dose, the odds of adverse cognitive outcome increased by 16% (95% CI 1.01–1.37; *p* = 0.04), independently of NE severity or parental educational level. Moreover, severe NE stage increased the odds of adverse cognitive outcome by 2.7 times (95% CI 1.32–7.06; *p* = 0.02) according to the adjusted model. The ROC curve analysis revealed that a cut-off value of 5.6 mg/kg cumulative HC dose predicts adverse cognitive outcome with 75.0% sensitivity and 68.3% specificity (AUC 0.71).

**Table 4:** Summary of the logistic regression modeling.

(a) Composite adverse outcome prediction model				
Variable	*β*	Odds ratio	95% CI	*p* value
Cumulative HC dose (mg/kg)	0.11	1.11	0.99–1.30	0.10
NE severity	0.71	2.03	1.14–4.00	**0.02**
Parental educational level	0.44	1.56	0.50–5.61	0.46
Nagelkerke R-squared: 0.27				

(b) Cognitive outcome predicition model				
Variable	*β*	Odds ratio	95% CI	*p* value
Cumulative HC dose (mg/kg)	0.15	1.16	1.01–1.37	**0.04**
NE severity	1.00	2.71	1.32–7.06	**0.02**
Parental educational level	−0.21	0.81	0.19–3.60	0.77
Nagelkerke R-squared: 0.38				

(c) Psychomotor outcome predicition model				
Variable	*β*	Odds ratio	95% CI	*p* value
Cumulative HC dose (mg/kg)	0.11	1.11	0.99–1.30	0.10
NE severity	0.71	2.03	1.14–4.00	**0.02**
Parental educational level	0.44	1.56	0.50–5.61	0.46
Nagelkerke R-squared: 0.27				

*n* = 47

*β* regression coefficient, *CI* confidence interval, *HC* hydrocortisone, *NE* neonatal encephalopathy.

Statistically significant P values are set in bold.

## Discussion

Data from this follow-up study of the randomized, controlled CORTISoL trial [4] demonstrated that the rate of composite adverse outcome was similar in the HC and placebo groups in patients with NE and systemic hypotension. Of note, beside the clinical severity of NE, the cumulative HC dose was also associated with adverse cognitive outcome, in an analysis that accounted for open-label HC, used as rescue therapy in a subset of patients.

Overall, it remains unclear whether glucocorticoids are neurotoxic or neuroprotective in neonatal hypoxic brain injury. Human data are still sparse, whereas animal models report conflicting results.

HC can cross the blood-brain barrier and binds preferentially to the mineralocorticoid receptors in the brain, although high concentrations may occupy both mineralocorticoid and glucocorticoid receptors. A region-specific difference in steroid receptor expression has been observed—mineralocorticoid receptors are predominantly localized in the limbic system, while glucocorticoid receptors are widely distributed throughout the brain, which may lead to altered local sensitivity to both exogenous and endogenous glucocorticoids. The neuronal effects of early steroid treatment may be long-lasting, site-specific, and conditional, and the overall action depends on the cellular context [19–21].

Cortisol (which is structurally identical to HC) is inactivated to cortisone by the 11-β-hydroxysteroid dehydrogenase type 2 enzyme, which is highly expressed in several regions of the neonatal brain [22]. This mechanism may help to protect against abundant mineralocorticoid and glucocorticoid receptor binding of exogenous corticosteroids. However, excessive HC doses and possible accumulation of the drug may saturate inactivating enzymes. Alternatively, hypoxic injury and/or hypothermia might possibly decrease enzyme function, causing increased activation of mineralocorticoid and glucocorticoid receptors. This could result in a dose-related undesirable side-effect of HC on the developing brain [23].

On the other hand, glucocorticoids may act as adjuvant therapy to hypothermia, because they can modulate apoptosis and inflammatory response to the hypoxic injury in the neonatal brain, and optimize neuroplasticity via glucocorticoid and mineralocorticoid receptor pathways based on animal hypoxic-ischemic encephalopathy models [23, 24].

Of note, evidence from clinical studies in preterm infants demonstrates adverse outcomes secondary to glucocorticoid therapy [5]. The use of early dexamethasone to prevent bronchopulmonary dysplasia in preterm neonates has been associated with abnormal brain development and increased risk of cerebral palsy [25]. Although comparative studies have shown that HC therapy has fewer short- and long-term adverse effects than dexamethasone therapy [26], some studies reported a higher rate of neurodevelopmental impairment in HC-treated preterm infants compared to placebo [10, 11]. Peeples et al. found that, in hypotensive preterm infants, low-dose HC had similar efficacy to high-dose HC with less adverse effects, supporting the proposal that clinicians should consider using the lowest effective HC dose. Additionally, hypotensive infants with baseline cortisol levels >15 µg/dl who received HC treatment had an increased rate of adverse side-effects, suggesting that HC therapy should be avoided in infants without relative adrenal insufficiency [27]. Similarly, Aucott et al. found that a higher serum cortisol level (independent of HC administration) was associated with adverse long-term outcomes in extremely low birth weight infants [28]. On the contrary, many clinical studies concluded that HC therapy was associated with similar [7–9, 26, 29, 30] or even better [6, 31] long-term outcome compared to placebo treatment.

In the present study, we found that early HC supplementation did not negatively influence the composite of severe neurodevelopmental impairment or death rates in infants with NE and hypotension. Although the statistical test did not show any difference between the HC and placebo groups, it should be noted that the rate of death or severe neurodevelopmental impairment was 40% in the HC group and 18% in the placebo group, which might be clinically relevant. Furthermore, in the multiple regression analysis, we showed that the cumulative HC dose is associated with the adverse cognitive outcome. Open-label HC administration was prompted by a worse clinical condition as a rescue therapy, which may explain at least in part this finding. Further studies would be required to prove or disprove the potential long-term side effects of HC in this patient population.

On the other hand, the timing of HC therapy may be an important determinant of neurodevelopmental outcome. While early HC administration after hypoxic injury may have neuroprotective effects [23], including promoting enhanced hemodynamic stability, it is conceivable that prolonged HC therapy beyond the first days after birth, or after hypothermia treatment may carry an increased risk for adverse neurodevelopmental outcome. Of note, in our clinical trial, the start of HC supplementation was median 11.5 h of life.

Despite the lack of convincing evidence for long-term benefit of HC therapy, the positive short-term effects on blood pressure have driven its continued and widespread use in hypotensive preterm and term neonates with impaired adrenal function and vasopressor-resistant hemodynamic instability [32, 33]. The lack of data on the pharmacokinetics of HC in neonates has resulted in a wide range of doses, with daily cumulative dosing that may vary up to 10 times depending on the center and guideline [32, 34, 35]. Moreover, the pharmacokinetic and pharmacodynamic properties of HC are unclear and may be further altered after major hypoxic damage and in hypothermic conditions [36, 37].

Beside the short-term hemodynamic benefit of using HC, in this study, we showed that the duration of cardiovascular therapy as well as peak dose of dopamine treatment were lower in the HC group compared to the placebo group. Furthermore, we previously demonstrated that longer cardiovascular therapy is independently associated with adverse long-term outcome in patients with NE [38]. Consequently, the use of HC, causing reduced vasopressor-inotrope exposure, may prevent further injury. It may be assumed that the excessive use of cardiovascular support, especially during rewarming, may increase the risk of reperfusion injury and long-term neurodevelopmental impairment.

There are limitations of the present study which require consideration. First, some infants received open-label HC therapy in the placebo group, as a rescue therapy due to refractory hemodynamic instability and/or critical deterioration. However, rescue supplementation started later, typically beyond the study period of therapeutic hypothermia. Second, the most severe cases were not enrolled, as infants with ongoing single or combined vasopressor-inotrope therapy were not included in this study. Future studies are needed to characterize the impact of HC in this high-risk population. Third, there may be additional post-discharge unaccounted determinants of neurodevelopmental outcome, although parental educational level, a major determinant of neonatal outcomes, was not different between the study groups. Fourth, we used the Bayley II test, which was the only validated version available in Hungary during the study period. This may reduce comparability with studies using Bayley III or IV, although conversion algorithms exist to support cross-version comparison [15, 39, 40]. Fifth, although statistical significance was not achieved when comparing the HC and placebo groups, the notable numerical difference in composite outcomes may still be clinically relevant, especially given the severity of these outcomes. Therefore, further investigation in larger studies is warranted.

In conclusion, in this small randomized trial, infants with NE and systemic low blood pressure were treated with HC in the early postnatal days and had neurodevelopmental outcomes at two years of age that were similar to the placebo group. Larger, multicenter trials focusing on long-term neurodevelopmental outcome are essential to prove or disprove the clinical utility and long-term effects of HC in this patient population.

## Supplementary information

Online Supplementary Material

Supplemental Figure 1

## Data Availability

The data that support the findings of this study are not openly available due to reasons of sensitivity and are available from the corresponding author upon reasonable request. Data are located in controlled access data storage at the Semmelweis University.
