# Supplementary material for: Neurodevelopmental outcome in infants with neonatal encephalopathy receiving hydrocortisone during therapeutic hypothermia: follow-up of the extended-CORTISoL trial
Source: J Perinatol. 2025 Sep 22;45(12):1788–94. doi: 10.1038/s41372-025-02428-5 (PMC12716990; doi:10.1038/s41372-025-02428-5)
Supplement: Supplementary file 1 — Online Supplementary Material [file 41372_2025_2428_MOESM1_ESM.docx]

**Clinical management**

Hypothermia was induced within 6 hours after birth and maintained for 72 hours using a servo controlled cooling device, Tecotherm Neo (Inspiration Healthcare, East Midlands, UK) targeting a rectal temperature range of 33 to 34°C. All infants received morphine infusion (10 μg/kg/h) (Morfina Cloridrato Monico, Monico SPA, Venezia/Mestre, Italy) and every patient was mechanically ventilated during the hypothermia treatment according to our institutional protocol. Phenobarbital (Gardenal, Sanofi Winthrop Industrie, France) was the first line anticonvulsant drug in cases of clinical and/or electrophysiological seizures.

When systemic low blood pressure was detected during hypothermia treatment one or two doses of fluid boluses (10 mL/kg per dose) were given to correct presumed hypovolemia. If volume expansion was deemed unsuccessful in maintaining blood pressure randomized treatment protocol was initiated beside standard dopamine administration. Hydrocortisone (HC) or placebo treatment was recommended to be continued until the end of rewarming period (as per the blinded treatment protocol), and in case of clinical deterioration HC treatment was continued until inotropes were weaned and hemodynamic stability was achieved. Some infants received open-label HC therapy, based on the decision of the attending neonatologist, irrespective of the randomization procedure, due to persistent high-dose vasopressor-inotrope requirements and/or critical deterioration and severe systemic hypotension.

Continuous single-channel biparietal amplitude-integrated electroencephalography (aEEG) (Olympic CFM 6000 Monitor; Olympic Medical, Seattle, WA, USA) was recorded until the end of rewarming period. The aEEG background activity was analyzed and scored for each 6-hour interval by a trained neonatologist (U.M.) blinded to the randomization, converting the Hellström-Westas criteria^1^ to a numeric value: continuous normal voltage (score 1), discontinuous normal voltage (score 2), burst suppression (score 3), continuous low voltage (score 4), and flat trace (score 5).^2^ Brain imaging was carried out at the Medical Imaging Center of Semmelweis University between the 1^st^ and 10^th^ postnatal day using a 3 Tesla Philips Achieva MR scanner (Philips Medical Systems, Best, The Netherlands). Routine non-contrast brain MRI protocols included: T1, T2, Diffusion Weighted Images and Apparent Diffusion Coefficient maps. We used the Weeke scoring system^3^ which is based on the assessment of injury to the grey matter (max. subscore of 23), cerebral white matter/cortex (max. subscore of 21) and cerebellum (max. subscore of 8). An additional subscore included the presence of intraventricular hemorrhage, subdural hemorrhage and sinovenous thrombosis (max. subscore of 3). The total score was calculated by adding the 4 subscores, resulting in maximum of 53. In our study, we analyzed the Weeke total score without including the MRS abnormalities. Severity of neonatal encephalopathy was ascertained by the attending physicians based on the overall aEEG background activity, occurrence of clinical and/or electrophysiological seizures, MRI results and clinical symptoms of encephalopathy.

Multiorgan failure was defined by failure of two or more organs with evidence of impaired function or altered physiology. Oliguria-anuria was defined by urine output <1 ml/kg/h for 12 hours during hypothermia treatment. Clinical illness severity was evaluated within 12 hours before the randomization using the Score for Neonatal Acute Physiology-II (SNAP-II). We calculated the scores from the lowest mean blood pressure (mmHg), lowest temperature (^o^C), PO2/FiO2 ratio, lowest pH, seizures and urine output (ml/kg/h).^4^

Serum cortisol levels were measured at randomization and thereafter at the time of other routine laboratory measurements in our patient population (at 6^th^, 24^th^, 48^th^ and 72^nd^ hours of life) by electrochemiluminescent immunoassays (Elecsys, Cobas E411; Roche, Basel, Switzerland). Cortisol levels remained blinded to the clinical team. The clinical management during hypothermia treatment was not influenced by the actual serum cortisol levels, as the clinicians did not see the results until the end of the study period.

**References**

1. Hellstrom-Westas L, Rosen I, Svenningsen NW. Predictive value of early continuous amplitude integrated EEG recordings on outcome after severe birth asphyxia in full term infants. Arch Dis Child Fetal Neonatal Ed 1995; 72:F34-8.

2. Meder U, Cseko AJ, Szakacs L, Balogh CD, Szakmar E, Andorka C, et al. Longitudinal Analysis of Amplitude-Integrated Electroencephalography for Outcome Prediction in Hypoxic-Ischemic Encephalopathy. J Pediatr 2022; 246:19-25 e5.

3. Weeke LC, Groenendaal F, Mudigonda K, Blennow M, Lequin MH, Meiners LC, et al. A Novel Magnetic Resonance Imaging Score Predicts Neurodevelopmental Outcome After Perinatal Asphyxia and Therapeutic Hypothermia. J Pediatr 2018; 192:33-40 e2.

4. Richardson DK, Corcoran JD, Escobar GJ, Lee SK. SNAP-II and SNAPPE-II: Simplified newborn illness severity and mortality risk scores. J Pediatr 2001; 138:92-100.
