# Supplementary figures and images for: Neurodevelopmental outcome in infants with neonatal encephalopathy receiving hydrocortisone during therapeutic hypothermia: follow-up of the extended-CORTISoL trial
Source: J Perinatol. 2025 Sep 22;45(12):1788–94. doi: 10.1038/s41372-025-02428-5 (PMC12716990; doi:10.1038/s41372-025-02428-5)

**Supplemental Figure 1.** Flow diagram.

**ENROLLMENT**

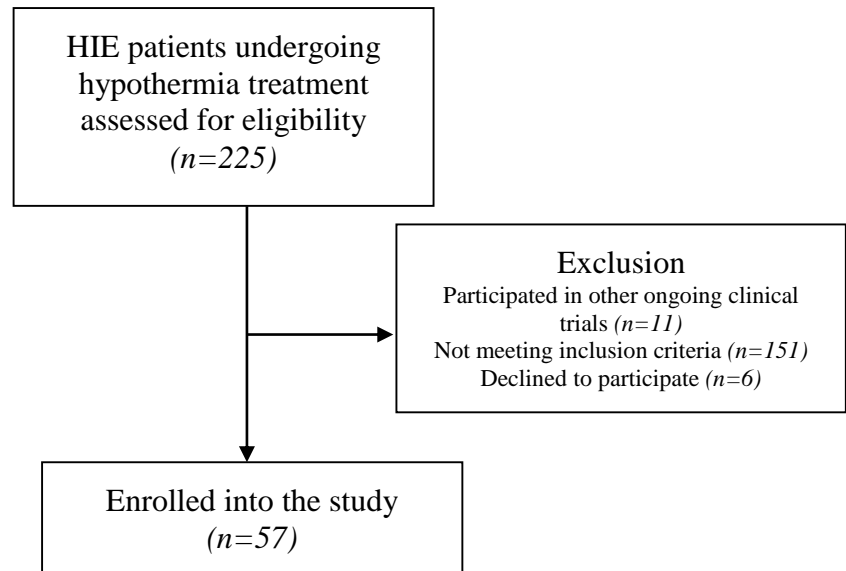

**ALLOCATION**

**ANALYSIS**

**FOLLOW-UP**

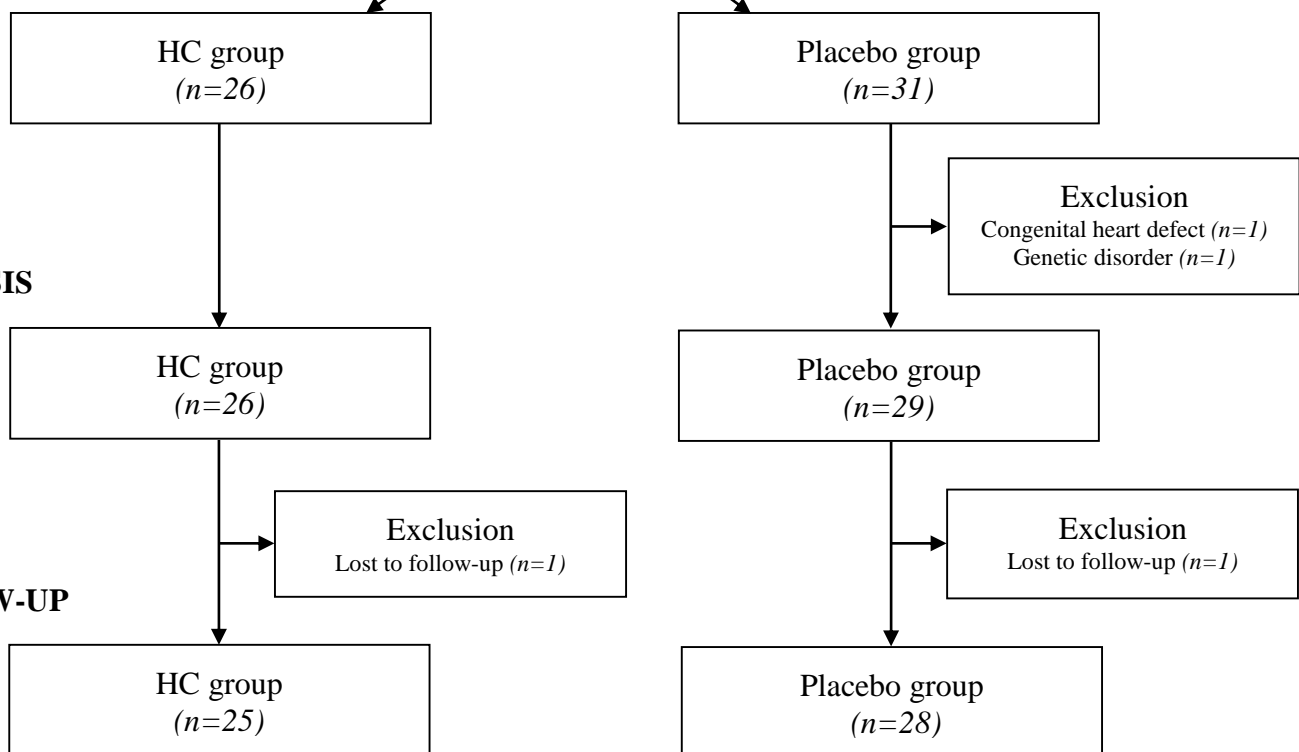

Supplement: Supplementary file 2 — Supplemental Figure 1 [file 41372_2025_2428_MOESM2_ESM.pdf]
